# A Review of NEPA, a Novel Fixed Antiemetic Combination with the Potential for Enhancing Guideline Adherence and Improving Control of Chemotherapy-Induced Nausea and Vomiting

**DOI:** 10.1155/2015/651879

**Published:** 2015-09-03

**Authors:** Paul J. Hesketh, Matti Aapro, Karin Jordan, Lee Schwartzberg, Snezana Bosnjak, Hope Rugo

**Affiliations:** ^1^Lahey Hospital & Medical Center, 41 Mall Road, Burlington, MA 01805, USA; ^2^Institut Multidisciplinaire d'Oncologie, Clinique de Genolier, Case Postale 100, Route du Muids 3, 1272 Genolier, Switzerland; ^3^Department of Internal Medicine IV, Hematology/Oncology, Martin-Luther-University Halle/Wittenberg, Ernst-Grube-Straße 40, 06120 Halle, Germany; ^4^The West Clinic, 100 Humphreys Boulevard, Memphis, TN 38120, USA; ^5^Institute for Oncology and Radiology of Serbia, Pasterova 14, 11000 Belgrade, Serbia; ^6^University of California San Francisco Helen Diller Family Comprehensive Cancer Center, 1600 Divisadero Street, P.O. Box 1710, San Francisco, CA 94115, USA

## Abstract

Combination antiemetic regimens targeting multiple molecular pathways associated with emesis have become the standard of care for prevention of chemotherapy-induced nausea and vomiting (CINV) related to highly and moderately emetogenic chemotherapies. Antiemetic consensus guidelines from several professional societies are widely available and updated regularly as new data emerges. Unfortunately, despite substantial research supporting the notion that guideline conformity improves CINV control, adherence to antiemetic guidelines is unsatisfactory. While studies are needed to identify specific barriers to guideline use and explore measures to enhance adherence, a novel approach has been taken to improve clinician adherence and patient compliance, with the development of a new combination antiemetic. NEPA is an oral fixed combination of a new highly selective NK_1_ receptor antagonist (RA), netupitant, and the pharmacologically and clinically distinct 5-HT_3_ RA, palonosetron. This convenient antiemetic combination offers guideline-consistent prophylaxis by targeting two critical pathways associated with CINV in a single oral dose administered only once per cycle. This paper will review and discuss the NEPA data in the context of how this first combination antiemetic may overcome some of the barriers interfering with adherence to antiemetic guidelines, enhance patient compliance, and offer a possible advance in the prevention of CINV for patients.

## 1. Introduction

The pathophysiology of chemotherapy-induced nausea and vomiting (CINV) is known to be a complex multifactorial process involving numerous neurotransmitters and receptors [1]. Consequently, combination antiemetic regimens targeting multiple molecular pathways associated with emesis have become the standard of care for prevention of CINV in patients receiving moderately (MEC) or highly emetogenic chemotherapy (HEC) [2–5]. The combination of a 5-HT_3_ receptor antagonist (RA) (targeting serotonin) and dexamethasone (DEX) represents the foundation of antiemetic prophylaxis for both MEC and HEC settings, with the addition of a neurokinin-1 (NK_1_) RA (targeting substance P), being uniformly recommended by antiemetic guidelines when administering HEC or anthracycline-cyclophosphamide (AC) chemotherapy [3–5].

Unfortunately, despite substantial research supporting the fact that guideline conformity will improve CINV control for patients, adherence to antiemetic guidelines is inadequate [6–9]. With the goal of improving the quality of care and quality of life for cancer patients undergoing emetogenic chemotherapy treatment, the Multinational Association of Supportive Care in Cancer (MASCC), in particular, continues to strive to educate clinicians on the importance and value of appropriate antiemetic prophylaxis. While studies are needed to identify specific barriers to guideline use within individual clinics and hospitals and to explore measures that can be taken to enhance adherence, an interesting approach has been taken with the development of a new combination antiemetic.

NEPA is an oral single dose, fixed combination agent, containing a new highly selective NK_1_ RA (netupitant) with the pharmacologically and clinically distinct 5-HT_3_ RA, palonosetron (PALO), thereby offering guideline-consistent prophylaxis while targeting two critical pathways associated with emesis. Palonosetron was selected for the combination over older generation 5-HT_3_ RAs due to its distinctive pharmacological properties [10, 11], its longer half-life compared with older 5-HT_3_ RAs, and its proven clinical efficacy [2, 12–14]. Its ability to work synergistically with netupitant suggests the potential to enhance prevention of delayed CINV when used in combination [10, 11].

This paper will briefly discuss the current antiemetic guideline recommendations and review the recently published NEPA data and discuss how this first combination antiemetic may overcome some of the barriers interfering with adherence to antiemetic guidelines and improve prevention of CINV for patients.

## 2. Updates to Antiemetic Guidelines, Importance of Adherence, and Consideration of Patient-Related Risk Factors

Evidence-based guidelines for the prevention of CINV have been developed by several international professional societies [MASCC, the European Society for Clinical Oncology (ESMO), and the American Society of Clinical Oncology (ASCO)]. The National Comprehensive Cancer Network (NCCN) in the United States has also developed antiemetic guidelines, and the same is true in many countries. These guideline committees meet regularly to review and discuss new data warranting revisions and updates to their recommendations [3–5]. While the guidelines of the various organizations vary to some extent, they are all reasonably consistent with their key recommendations (Table 1).

It is important to realize that antiemetic guideline committees continue to group their recommendations based on the emetogenicity of the chemotherapy, notwithstanding awareness of well-established patient-related risk factors that increase patients' emetic risk. These risk factors include female gender, younger age, history of low alcohol intake, motion sickness, experience of emesis during pregnancy, anxiety, impaired performance status, and previous exposure to chemotherapy [15–18].

Patients receiving treatment with a combination of an anthracycline and cyclophosphamide may present a particularly challenging population not only due to the intrinsic emetogenicity of this chemotherapy combination but also because AC is commonly used in young, female breast cancer patients. These patient-related factors of female gender and younger age can add to the emetogenicity of the chemotherapy. Recently, guideline groups either established a separate category/recommendation for AC chemotherapy (MASCC/ESMO) or reclassified AC from the previous category of being moderately emetogenic to being highly emetogenic (ASCO/NCCN). Regardless of the specific approach to classification, all committees now recommend that patients receiving AC should receive the triplet combination of an NK_1_ RA plus 5-HT_3_ RA plus dexamethasone (Table 1). Whether or not guideline committees integrate patient-related risk factors with chemotherapy emetogenicity, clinicians need to give patient risk factors consideration when determining the optimal antiemetic prophylaxis for a given patient [16].

Despite the fact that antiemetic guidelines are widely available and data supports the notion that guideline conformity improves CINV control for patients [6, 7], clinical utilization of guidelines remains unacceptably low. Recently, Aapro and colleagues showed guideline adherence of only 29% in a large 1000-patient European observational study [6]. Better CINV control and less utilization of health care resources were also observed in patients receiving guideline-consistent antiemetic prophylaxis, suggesting a clear need for greater adherence to the guideline recommendations. In a subsequent study conducted in US oncology practices, Gilmore and colleagues similarly showed adherence to NCCN guidelines to be low, particularly in patients receiving HEC (29% adherence HEC, 73% in MEC) [7]. As in the Aapro study, adherence to guidelines was associated with significantly better CINV control in HEC and MEC settings. In a recent analysis of IMS Health Inc. data from 5 European countries between January and December 2013, only about 12% of patients receiving HEC, 14% of patients receiving AC, and 47% of patients receiving non-AC MEC were prophylactically administered antiemetics in accordance with the MASCC/ESMO guidelines [19].

Inadequate adherence to practice guidelines is not specific to antiemetics; it is common across all fields of medicine with numerous factors playing a role. A key aspect related to utilizing guidelines is the behavior of the clinician, and often changing behavior is difficult. Physician knowledge, clinician and institutional education, attitudes toward guidelines, clinician agreement with them, awareness of and familiarity with them, lack of confidence in their ability to implement them, and expectations may all impact use [20–22]. In addition, in many countries local regulations do not allow access to antiemetic agents recommended by international guidelines. Only a small number of studies have evaluated approaches to improving adherence with antiemetic guidelines, and some were hindered by methodological shortcomings [23–26]. Nevertheless, some important messages can be derived from these studies. Single approaches to addressing adherence have little, if any, impact [21]. Multiple strategies need to be used concurrently in order to improve adherence and implementation of antiemetic guidelines. These can include guideline dissemination, use of opinion leaders, interactive educational workshops, therapeutic reminders in the form of preprinted orders, clinical interventions by pharmacists for inappropriate antiemetic orders, and physician audit and feedback. A key approach appears to be communication of patients' CINV outcomes to physicians. Patient-mediated approaches and computerized decision-support systems may be promising approaches to be utilized in the future, possibly in combination with the multifaceted strategies described above [16]. A practical approach to better implementation of the guidelines is crucially needed to improve antiemetic care and outcomes for patients undergoing emetogenic chemotherapy.

## 3. NEPA Pharmacology

Netupitant is a highly selective NK_1_ RA with a high degree of receptor occupancy. A positron emission tomography (PET) study showed NK_1_ receptor occupancy ≥90% in the majority of the brain regions tested at *C*_max⁡_, with a long duration of receptor occupancy at doses of 100–450 mg. The netupitant minimal plasma concentration predicted to achieve an NK_1_ RA of 90% in the striatum was 225 *μ*g/mL. Netupitant 300 mg was the lowest oral dose reaching this value [27].

Palonosetron is a “new-generation” 5-HT_3_ RA with a longer half-life and distinct pharmacological properties compared with older agents in the 5-HT_3_ RA class. Mechanism of action studies have shown that unlike other 5-HT_3_ RAs, palonosetron exhibits allosteric interactions, positive cooperativity, and persistent inhibition of receptor function; it also triggers receptor internalization and inhibits signaling crosstalk between 5-HT_3_ and NK_1_ receptors [10]. Most recently,* in vitro* studies have shown that the combination of netupitant and palonosetron exhibits a synergistic effect in preventing the NK_1_ receptor response against its endogenous agonist, substance P [11], and an additive effect on NK_1_ receptor internalization [28]. The plasma elimination half-lives of palonosetron (>40 hours) and netupitant (~96 hours) are long, likely contributing to the extended efficacy during the delayed phase (25–120 hours) following chemotherapy administration [10].

Netupitant is a substrate and moderate inhibitor of the cytochrome P450 isoenzyme 3A4 (CYP3A4) and therefore, as is the case with another NK_1_ RA, aprepitant, coadministration with drugs that are substrates of CYP3A4 may require dose adjustments [29–31]. Notably, the dose of dexamethasone should be reduced when used in combination with NEPA [30]; this was done in the NEPA clinical trials. However, unlike aprepitant, netupitant does not result in clinically relevant interactions with oral contraceptives, and interactions with CYP2C9 substrates (e.g., warfarin, tolbutamide) are unlikely based on* in vitro* interaction data [31]. While the potential for netupitant interactions with chemotherapy agents metabolized by CYP3A4 has not been fully established, no interaction or no clinically relevant interaction has been observed between aprepitant and the commonly administered chemotherapeutic agents (cyclophosphamide, docetaxel, and intravenous vinorelbine) [32]. Other agents that are known to be metabolized by CYP3A4 include paclitaxel, etoposide, irinotecan, ifosfamide, imatinib, vinblastine, and vincristine.

## 4. NEPA Efficacy in Prevention of CINV

### 4.1. Overview of Studies

The efficacy of NEPA has been evaluated in 3 pivotal registration trials, all in chemotherapy-naïve patients with predominantly solid tumors receiving a variety of highly and moderately emetogenic chemotherapeutic agents (Table 2).

Two studies (07-07 [Study 1] and 08–18 [Study 2]) were designed to demonstrate superiority of NEPA over oral palonosetron. Study 1 was a pivotal, dose-ranging trial designed to identify the best dose combination for NEPA in patients receiving cisplatin-based HEC. Study 2 was designed to show superiority of the selected NEPA dose over oral palonosetron in patients receiving AC. While the third study (10–29 [Study 3]) in patients receiving a variety of HEC and MEC (excluding breast cancer patients receiving AC) was designed primarily to evaluate the safety of NEPA over multiple cycles, efficacy was also assessed and described. This study included an aprepitant treatment arm; however, as the inclusion of this arm was intended to help interpret any unexpected safety finding, no formal efficacy comparisons were prospectively planned and performed.

In all 3 trials a single dose of NEPA was administered 60 minutes prior to chemotherapy on Day 1. Oral palonosetron (Studies 1 and 2) and aprepitant (Studies 1 and 3) were administered at this same time on Day 1; aprepitant was also given in the morning of Days 2 and 3. Dexamethasone was administered 30 minutes prior to chemotherapy on Day 1 and according to the MASCC/ESMO antiemetic guideline recommendations (i.e., administered on Days 1–4 for patients receiving HEC and on Day 1 only in patients receiving MEC). The dexamethasone doses with NEPA (and aprepitant) were 12 mg PO on Day 1 and additionally 8 mg on Days 2–4 in the HEC setting. The dexamethasone doses with palonosetron were 20 mg on Day 1 and 16 mg on Days 2–4 (with HEC). Blinding of treatment groups was maintained in all studies with the use of matching identical placebos.

The primary efficacy endpoint of interest was proportion of patients with a complete response (CR: no emesis and no rescue medication). Other efficacy endpoints included proportion of patients with no emesis, no significant nausea [defined as a maximum score of <25 mm on a 100 mm visual analog scale (VAS)], and complete protection (CR + no significant nausea). In the Phase 3 NEPA superiority trial (Study 2), patients also completed a functional living index emesis (FLIE) questionnaire, a validated 18 item VAS-based, patient-reported outcome measure that assesses the impact of CINV on patients' daily lives/functioning. All efficacy endpoints were evaluated during the acute (0–24 h), delayed (25–120 h), and overall (0–120 h) phases after chemotherapy administration. Details of the study designs, inclusion/exclusion criteria, patient demographics, and statistical analyses are reported in the individual publications [33–35].

### 4.2. Dose Selection

Study 1 was a phase 2, pivotal, dose-ranging trial in 694 patients receiving cisplatin-based chemotherapy. It was designed to evaluate 3 different oral doses of netupitant (100, 200, and 300 mg) coadministered with oral palonosetron 0.50 mg to determine the most appropriate clinical dose for the NEPA combination [33]. The 0.50 mg oral palonosetron dose was selected as it represents the approved oral dose of palonosetron [36]. While all coadministered doses provided superior prevention of CINV compared with oral palonosetron, netupitant 300 mg + palonosetron 0.50 mg was the best combination dose when considering all efficacy endpoints. There was no difference in safety between doses of netupitant. The 300 mg netupitant dose was also the minimal dose tested in the PET study resulting in receptor occupancy of 90% in the striatum [27]. This level of receptor occupancy has been used historically in studies with aprepitant to predict antiemetic efficacy.

This oral fixed combination of 300 mg netupitant + 0.50 mg palonosetron was subsequently developed and evaluated in the NEPA Phase 3 clinical development program.

### 4.3. Cycle 1 Efficacy

**Figure 1:**
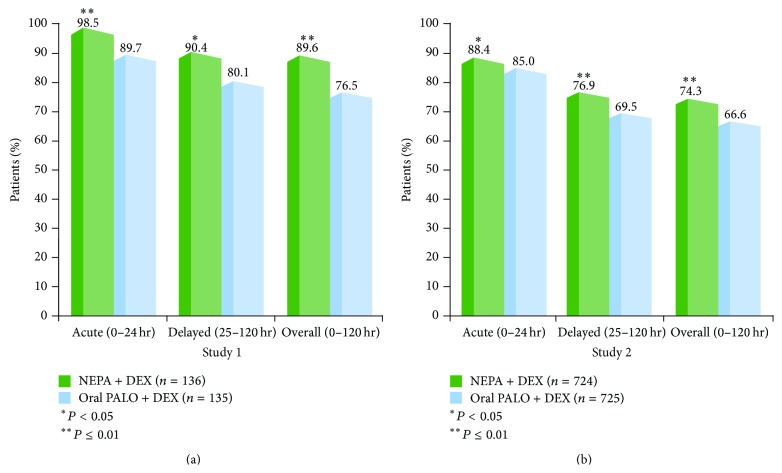
Cycle 1 complete response (no emesis, no rescue medication) rates: NEPA versus oral palonosetron (Studies 1 and 2).

In both of the trials comparing NEPA and oral palonosetron (Studies 1 and 2), NEPA showed superior prevention of CINV during the overall phase as demonstrated by significantly higher CR rates (Figure 1) as well as absence of emesis, absence of significant nausea, and complete protection rates (Table 3) [33, 34]. Superiority of NEPA over oral palonosetron was also seen for the same four efficacy endpoints during the delayed phase in both trials. During the acute phase, NEPA was superior to oral palonosetron for all endpoints in the HEC study and for CR and no emesis in the AC-MEC study (Figure 1; Table 3).

In Study 2, this better prevention of both nausea and vomiting correlated with a quality-of-life benefit for patients. For the FLIE assessment, significantly more NEPA-treated patients (79%) reported no impact on daily functioning for the total combined domains of nausea and vomiting during the 5 days after chemotherapy compared with those treated with oral palonosetron (72%; *P* = 0.005) [34]. A significantly greater proportion of NEPA-treated patients also had no impact on functioning due specifically to nausea (72% NEPA versus 66% oral PALO, *P* = 0.015) and due specifically to vomiting (90% NEPA versus 84% oral PALO, *P* = 0.001).

In the Phase 3 safety study (Study 3), the overall CR rates for NEPA in Cycle 1 were high; 81% for the total population, 84% in the subgroup of patients receiving HEC, and 80% for the subgroup of patients receiving MEC [35, 37]. Similar results were seen with the proportion of patients with no significant nausea (84% overall population, 82% HEC subgroup, and 85% MEC subgroup) [37].

#### 4.3.1. Efficacy in Gender/Age Risk Subgroups

Female gender and young age are well-established patient-related risk factors increasing the emetogenic potential of chemotherapy. In order to evaluate the effect of gender and age on treatment response, data was combined from Phase 2 and 3 comparative trials 1 and 2 as well as a third trial which included oral palonosetron [38, 39]. Overall CR rates were calculated for females and males and for patients < 55 years and ≥ 55 years.

As expected, in both NEPA and oral palonosetron treatment groups, overall CR rates were numerically lower in females (82% NEPA, 69% oral PALO) compared with males (91% NEPA, 78% oral PALO) and also lower in those < 55 yrs (85% NEPA, 70% oral PALO) compared with those ≥ 55 yrs (89% NEPA, 77% oral PALO), although no formal statistical comparison was performed. However, the beneficial effect of NEPA over oral palonosetron was seen in both gender and age groups as evidenced by a similar absolute difference of 12–15%.

To evaluate the combined effect of gender plus age, patients were divided into 4 emetic risk groups (females < 55 years [high risk], females ≥ 55 years [moderate risk], males < 55 years [low risk], and males ≥ 55 years [lowest risk]). A clear trend existed across the risk groups with (older) males exhibiting numerically higher CR rates than (younger) females and CR rates numerically higher for NEPA than oral palonosetron in all gender/age risk groups (Table 4).

#### 4.3.2. Efficacy in Older Patients

Prevention of CINV in older cancer patients is critical, as these patients tend to be more sensitive to the adverse effects of cytotoxic therapy and thus more likely to experience dehydration and anorexia related to CINV [40, 41]. NEPA data from the 3 pivotal trials was combined to evaluate the efficacy in an older subgroup of patients (*n* = 214 ≥65 years old) [42]. CR rates for NEPA in the older patients were generally higher than those seen in the overall study population (Table 5).

#### 4.3.3. Efficacy in Patients Receiving Cisplatin Plus Concomitant Chemotherapy

It has been previously shown that antiemetic efficacy is reduced when concomitant emetogenic chemotherapy is administered concurrently with cisplatin [43]. To evaluate whether emetic prevention differed for NEPA with the addition of concomitant chemotherapy, NEPA groups from the Phase 2 dose-ranging trial (Study 1) were combined [44]. Complete response and no significant nausea rates were then calculated for two groups of patients: those receiving cisplatin plus no/minimal/low emetic risk chemotherapy or those receiving cisplatin plus moderate/high emetic risk chemotherapy.

CR and no significant nausea rates were similar for the acute, delayed, and overall intervals for both groups, regardless of the emetogenicity of the additional chemotherapy administered with cisplatin.

Overall (0–120 h) CR rates were 88% and 87% for the lower and higher emetic risk groups, respectively, while no significant nausea rates were 86% and 85%, respectively.

#### 4.3.4. Efficacy in Patients Receiving Carboplatin

As there is limited data supporting a guideline recommendation for the addition of an NK_1_ RA to a 5-HT_3_ RA/DEX regimen with platinum agents other than cisplatin, a post hoc analysis from Study 3 was performed to assess the effectiveness of NEPA in 149 patients receiving carboplatin [45]. The overall CR rates for NEPA were 80%, 91%, 92%, and 94% for cycles 1–4, respectively. Similar results were seen for no emesis, with rates of 83%, 91%, 92%, and 95% for cycles 1–4, respectively.

### 4.4. Multiple Cycle Efficacy

Most antiemetic trials assess CINV control in only a single cycle of treatment. However, preservation of benefit over repeated cycles of chemotherapy is essential for optimal supportive care during cancer treatment. Two studies in the NEPA clinical program evaluated the effectiveness of NEPA over multiple cycles of chemotherapy. The Phase 3 Study 2 in patients receiving AC comparing NEPA with oral palonosetron included a multiple cycle extension [46]. The multiple cycle safety Study 3 also assessed efficacy over cycles [35].

**Figure 2:**
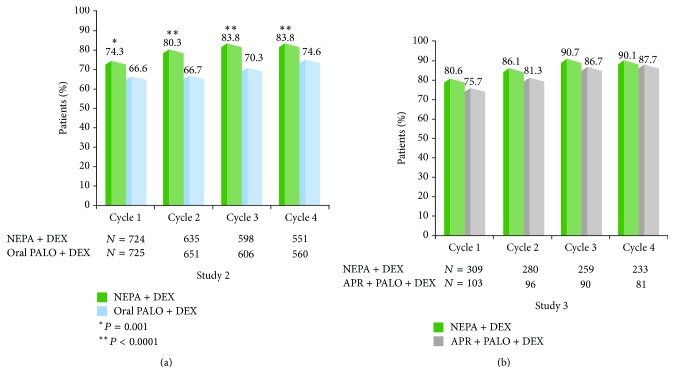
Overall (0–120 h) complete response (no emesis, no rescue medication) rates over multiple chemotherapy cycles: NEPA versus oral palonosetron (Study 2) and NEPA versus aprepitant regimen (Study 3).

1033 NEPA-treated patients participated in 4428 total chemotherapy cycles in these two trials; 75% of patients completed at least 4 cycles. In Study 2, the proportion of patients with an overall CR was significantly greater for NEPA compared with oral palonosetron during cycles 1–4 (Figure 2(a)) [46]. NEPA was also significantly more effective than oral palonosetron in preventing no emesis and no significant nausea over cycles 1–4. While no formal efficacy comparisons with aprepitant were intended in Study 3, the overall CR rates were high and were maintained across cycles for both NEPA and the aprepitant/palonosetron/DEX regimen, with NEPA showing a small but consistent numerical advantage (2%–7%) over aprepitant during each cycle (Figure 2(b)) [35]. Response rates for NEPA were similar in the subgroups of patients who received HEC and non-AC MEC. Similar results were seen for no significant nausea in the overall population as well as the emetogenicity subgroups of HEC and MEC.

### 4.5. Comparison with Aprepitant Regimen

An aprepitant/5-HT_3_ RA/DEX regimen was included for exploratory purposes in the dose-ranging trial (Study 1) and to help interpret any unexpected safety finding in the multiple cycle HEC/MEC Study 3. In Study 1, Hesketh and colleagues reported that the aprepitant/ondansetron/DEX arm showed higher CR and no emesis rates compared with oral palonosetron during the overall and delayed phases, but not the acute phase [33]. It also resulted in numerically higher no significant nausea and complete protection rates, but these were not significantly different from oral palonosetron during any time interval after chemotherapy. Although no formal comparisons were performed and the differences were small, the NEPA combination selected for development had numerically higher response rates than the multiday aprepitant regimen for all efficacy endpoints and time intervals. As mentioned previously, while no formal efficacy comparisons were performed, NEPA also showed numerically higher CR rates than the aprepitant/palonosetron/DEX regimen over multiple cycles in Study 3 [35].

## 5. Safety of NEPA

In each of the individual studies, the overall incidence, type, frequency, and intensity of treatment-emergent adverse events was as expected for the 5-HT_3_ RA and NK_1_ RA classes and for patients undergoing cytotoxic chemotherapy. NEPA had a similar adverse event profile to oral palonosetron and the aprepitant-based regimen [33–35]. The most frequent treatment-related adverse events were headache and constipation. Aapro et al. [47] presented a comprehensive overview of the safety of NEPA, pooling data from the studies in the development program. The percentages of patients with at least 1 treatment-emergent adverse event (TEAE) in Cycle 1 and in all cycles were generally similar for NEPA, oral palonosetron, and the aprepitant groups as were the percentages of patients reporting AEs considered to be treatment-related (Table 6). Few patients in any group experienced serious AEs or AEs leading to discontinuation or death. There were no deaths in the clinical program considered to be related to the NEPA treatment.

A similar frequency of cardiac AEs was reported in each treatment group during all cycles of treatment [47]. The mean changes from baseline in the ECG parameters assessed (heart rate, PR, QRS, QT, QTcB, and QTcF) were small and generally similar across the treatment groups at each study time point. Neither netupitant or oral palonosetron has shown any signals for effects on corrected QT interval (QTcl), heart rate, PR, or QRS intervals compared to placebo in separate ICH E14 QT trials in healthy volunteers [48, 49]. This has also been shown for palonosetron in studies in patients with cancer [50–52].

## 6. Discussion and Conclusions

With the introduction of the NK_1_ RA class into the antiemetic armamentarium, the last decade of research has focused on better understanding the pathophysiology of CINV and identifying effective antiemetic combinations which target multiple molecular pathways associated with emesis. Accordingly, CINV can now be prevented and/or minimized successfully for the majority of patients. However, this control can only be achieved if appropriate antiemetic prophylaxis is administered to patients.

While there are some differences between the various antiemetic guidelines, they all provide evidence-based reasonably consistent recommendations to guide clinicians on the optimal use of antiemetics. Unfortunately, barriers exist which continue to interfere with administration of guideline-based antiemetic prophylaxis [6–9, 20–22], despite compelling research indicating that nonadherence leads to diminished CINV control for patients [6, 7]. In the large studies by Aapro and Gilmore [6, 7], it was discouraging that the patients at highest risk for CINV (i.e., those receiving HEC or AC chemotherapy) were the ones where the incidence of guideline inconsistent prophylaxis was highest. As these are the patients most likely to benefit from appropriate antiemetics, it is critical that continued efforts are taken to identify multifaceted strategies that can be employed to improve guideline adherence. The Aapro study also suggested that the absence of an NK_1_ RA in these high risk groups was a predominant gap in guideline-consistent care. While economic constraints of hospitals and government payers may have contributed to the underutilization of the NK_1_ RA, the complexity and inconvenience of the oral aprepitant regimen (e.g., 3 days aprepitant plus 1–3 days 5-HT_3_ RA plus 1–4 days of dexamethasone) may have also played a role. In light of this, it is interesting to speculate if the development of the convenient single day antiemetic combination, NEPA, will offer physicians a possible solution to address this particular gap.

The data in the clinical program offer unequivocal evidence that NEPA plus dexamethasone provides superior CINV control over oral palonosetron plus dexamethasone in settings where an NK_1_ RA/5-HT_3_ RA/DEX “triplet” is recommended (i.e., HEC and AC-based MEC). This is supported by the pharmacological synergy seen with netupitant and palonosetron. The consistent superiority of NEPA over oral palonosetron across the multiple efficacy endpoints in two comparative studies is particularly noteworthy considering that palonosetron is regarded as a clinically distinct 5-HT_3_ RA.

Future trials could be considered to explore how NEPA compares clinically to an aprepitant-based triplet regimen. The limited data generated thus far suggests that NEPA shows slightly higher response rates than a 3-day aprepitant regimen in both the single cycle study in patients receiving cisplatin-based HEC (Study 1) [33] and over multiple cycles in patients receiving either MEC or HEC (Study 3) [35]. However, as neither of these trials were designed specifically to compare the efficacy of NEPA with that of the aprepitant regimen, a formal trial would be needed to address this.

Study 3 offers evidence that NEPA is effective in a non-AC-based “pure” MEC population, although further studies are needed to evaluate the superiority of NEPA over a 5-HT_3_ RA/DEX regimen in this setting.

With the appropriate use of current antiemetics, emesis can be prevented in nearly all patients. However, there is still room for improving control of nausea, particularly during the delayed phase following chemotherapy. As no consistent superiority has been seen with the addition of aprepitant over 5-HT_3_ RAs + DEX in previous trials evaluating nausea [53–57], it was encouraging to see that a nausea benefit was demonstrated with NEPA over oral palonosetron in two pivotal trials. High levels of protection against nausea were also maintained in multiple cycles (i.e., in the extension of the Study 2 and also in Study 3) (Table 3). Notwithstanding this benefit, further research is needed to better understand nausea and how to optimize control of this bothersome symptom, particularly in subgroups of patients who may be more prone to it.

In the subset of older patients ≥65 years, it was reassuring to see that CINV control with NEPA was at least as good as that seen in the overall population in the individual pivotal trials. In the gender/age analysis, it was not surprising that males had higher CR rates than females as did older patients compared with younger patients. It was somewhat surprising, however, that despite these differences in response by gender and age the beneficial effect of NEPA over oral palonosetron was consistent in males and females (13% in both) and in older (12%) and younger (15%) patients. This is in contrast to previously reported aprepitant data where the beneficial effect of an aprepitant/ondansetron/DEX regimen over ondansetron/DEX was greatest in women (14%) and in those < 55 years (19%) compared with men (4%) and those ≥ 55 years (6%) [58]. It is encouraging that NEPA offers an improved response regardless of age and gender and also regardless of the combined risk factors of young age/female (high risk) versus older/males (lowest risk) where the NEPA benefit seen ranged from 11% to 18%.

The data in the subset of 149 patients receiving carboplatin is interesting. The response rates are consistent with a similar historical carboplatin subset analysis and more recently a Phase 3 trial in patients receiving a paclitaxel/carboplatin regimen where an aprepitant regimen showed a >10% incremental benefit over a 5-HT_3_ RA/DEX control [59, 60]. Given this consistent evidence, MASCC/ESMO and ASCO guideline groups, in particular, should evaluate this dataset and emerging data with rolapitant [61] and give consideration to the addition of an NK_1_ RA in patients receiving carboplatin. NCCN includes carboplatin as one of the chemotherapies, like AC, where an NK_1_ RA should be added to the 5-HT_3_ RA/DEX regimen (Table 1).

The multiple cycle data generated for NEPA is perhaps the most robust multicycle dataset generated thus far for any approved antiemetic. In Studies 2 and 3, 1033 patients were treated with NEPA for a total of 4428 chemotherapy cycles with 75% of these patients completing at least 4 cycles [37]. Overall CR and no significant nausea rates were high and were maintained across 4 cycles of chemotherapy in both studies and in the subsets of patients receiving the various types of chemotherapies. This provides confidence in the preservation of benefit with NEPA over repeated cycles and reinforces the value of administering appropriate prophylaxis starting at Cycle 1, rather than waiting to introduce an NK_1_ RA after the patient has failed (i.e., experienced CINV).

The safety profile for NEPA is consistent with that expected for these drug classes with the type and incidence of adverse events also being typical for a diverse cancer population receiving cytotoxic chemotherapy. As expected, the most common treatment-related adverse events were headache and constipation. While there has been safety concerns associated with QTc prolongation with older 5-HT_3_ RAs such as ondansetron and dolasetron [62–64], the cardiac adverse events and QTc data for NEPA from the pivotal studies indicate no cardiac safety concerns. Reassuringly, neither netupitant nor palonosetron have shown any signals for effects on corrected QT intervals in individual QT trials [48–52].

Because netupitant is a moderate inhibitor of CYP3A4, the oral dexamethasone dose should be reduced when used in conjunction with NEPA. The dexamethasone doses administered in the NEPA clinical trials were 12 mg PO on Day 1 (for HEC and AC) and additionally, 8 mg on Days 2–4 in the HEC setting. The administration of dexamethasone on Day 1 only in the AC trial was consistent with the MASCC/ESMO guideline recommendations. In contrast, the ASCO and NCCN guidelines recommend dexamethasone dosing through Day 3 or 4 in the AC setting. However, as dexamethasone may be associated with a range of side effects, there is interest in minimizing its dose and frequency, particularly in those patients who experience dexamethasone-related side effects or in those with preexisting conditions that may be exacerbated by corticosteroid use. The NEPA data in Study 2 provide encouraging evidence that a Day 1 only regimen of NEPA plus dexamethasone is effective. As concomitant administration of NEPA may increase the plasma concentration for drugs that are mainly metabolized via CYP3A4, coadministration with other drugs that are substrates of CYP3A4 may require dose adjustments. While these potential drug-drug interactions were a possible source of concern regarding aprepitant, a review by Aapro and colleagues concluded that the majority of drug-drug interactions with aprepitant have little or no clinical consequence [32]. Reassuringly, in the large Phase 3 study where all patients received cyclophosphamide, an alkylating agent metabolized in part via CYP3A4, a similar AE profile was seen for NEPA and palonosetron, with no increased incidence of AEs that would be typically associated with cyclophosphamide [65, 66].

As with any new drug, the registration trials offer the critical information needed for approval and to assist physicians in understanding the overall benefit of the new treatment. With this new and novel combination antiemetic, further research will be necessary to understand its place and benefit in preventing CINV in settings where patients are receiving multiday chemotherapy and in patients with hematologic malignancies. In addition, while the benefit of NEPA over a 5-HT_3_ RA/DEX regimen is clear in patients at higher risk for CINV, it will be helpful to understand if this combination can offer a benefit to a broader group of patients receiving other moderately emetogenic chemotherapy.

NEPA has the potential to overcome some of the barriers hindering antiemetic guideline adherence by packaging guideline-consistent antiemetic prophylaxis in a single, oral dose that is taken only once per cycle. Evaluation of whether this translates into improved patient compliance and guideline adherence and potentially reduced nursing time, clinic/emergency room visits, and follow-up care will be important to assess in clinical practice.

## Figures and Tables

**Table 1:** Key recommendations of antiemetic guideline groups.

Emetic risk category	MASCC/ESMO (2010) [3]		ASCO (2011) [4]		NCCN (2014) [5]	
	Day 1	Days 2-3	Day 1	Days 2-3	Day 1	Days 2-3
High	NK_1_ RA + 5-HT_3_ RA + DEX	NK_1_ RA^a^ + DEX	Same as MASCC	Same as MASCC	Same as MASCC^c^ or olanzapine + PALO + DEX	NK_1_ RA^a^ + DEX^d^ or olanzapine^e^

AC	NK_1_ RA + 5-HT_3_ RA + DEX	NK_1_ RA^a^	Same as MASCC^b^	DEX + NK_1_ RA^a^	Same as MASCC^c^ or olanzapine + PALO + DEX	Same as MASCC^d^ or olanzapine^g^

Moderate	PALO + DEX	DEX	Same as MASCC	Same as MASCC	Same as MASCC^c^ or NK_1_ RA + 5-HT_3_ RA + DEX^f^ (in select patients)	5-HT_3_ RA^h^ or DEX

Low	DEX or 5-HT_3_ RA or DRA	No routine prophylaxis	DEX	Same as MASCC	Same as MASCC^i^	Same as MASCC

Minimal	No routine prophylaxis	No routine prophylaxis	Same as MASCC	Same as MASCC	Same as MASCC	Same as MASCC

^a^NK_1_ RA (aprepitant) is given only if aprepitant was given on Day 1; if fosaprepitant was used then no follow-up NK_1_ RA is administered.

^b^AC is classified as highly emetogenic.

^c^Palonosetron is preferred 5-HT_3_.

^d^Given on Days 2–4 (i.e., an additional day).

^e^If olanzapine regimen was given on Day 1.

^f^As per highly emetogenic recommendations an NK_1_ regimen should be administered with certain MEC agents (e.g., carboplatin, doxorubicin, epirubicin, ifosfamide, irinotecan, and methotrexate).

^g^If olanzapine was given on Day 1.

^h^Only an option if a 5-HT_3_ other than PALO was used on Day 1.

^i^Specifically metoclopramide or prochlorperazine.

AC: anthracycline cyclophosphamide; NK_1_ RA: neurokinin 1 receptor antagonist; 5-HT_3_ RA: serotonin receptor antagonist; DEX: dexamethasone; DRA: dopamine receptor antagonist; PALO: palonosetron.

**Table 2:** NEPA study designs.

Study	Study design	Patient population/chemotherapy	Treatment groups	Single versus multiple cycle	Study objective
**Study 1** [Hesketh et al.] [33]	Double-blind, randomized, dose-ranging, parallel group Phase 2 (*N* = 694)	Chemotherapy-naïve Cisplatin-based chemotherapy (HEC)	NEPA_100_ + DEX NEPA_200_ + DEX NEPA_300_ + DEX Oral PALO + DEX APR + IV OND + DEX (included as exploratory)	Single cycle	Identify best dose of NETU + PALO; demonstrate superiority of NEPA over oral PALO

**Study 2** [Aapro et al.] [34]	Double-blind, randomized, parallel group Phase 3 (*N* = 1455)	Chemotherapy-naïve Anthracycline-cyclophosphamide	NEPA + DEX Oral PALO + DEX	Multiple cycle	Demonstrate superiority of NEPA over oral PALO

**Study 3** [Gralla et al.] [35]	Double-blind, randomized 3 : 1, parallel group Phase 3 (*N* = 413)	Chemotherapy-naïve Any HEC or MEC (except AC)	NEPA + DEX APR + oral PALO + DEX (3 : 1 randomization)	Multiple cycle	Demonstrate multiple cycle safety and describe efficacy of NEPA

HEC: highly emetogenic chemotherapy; MEC: moderately emetogenic chemotherapy; AC: anthracycline cyclophosphamide; DEX: dexamethasone; PALO: palonosetron; NETU: netupitant; APR: aprepitant; OND: ondansetron; NEPA_100_: NETU 100 mg + oral PALO 0.50 mg; NEPA_200_: NETU 200 mg + oral PALO 0.50 mg; NEPA_300_: NETU 300 mg + oral PALO 0.50 mg; IV: intravenous.

**Table 3:** Cycle 1 Efficacy of NEPA + DEX compared with oral palonosetron + DEX.

Patients (%)	Study 1 (Cisplatin HEC)			Study 2 (AC)		
	NEPA + DEX (*N* = 136)	Oral PALO + DEX (*N* = 135)	*P* value^1^	NEPA + DEX (*N* = 724)	Oral PALO + DEX (*N* = 725)	*P* value^2^
**No emesis**						
Acute (0–24 h)	98.5	89.7	0.007	90.9	87.3	0.025
Delayed (25–120 h)	91.9	80.1	0.006	81.8	75.6	0.004
Overall (0–120 h)	91.1	76.5	0.001	79.8	72.1	<0.001
**No significant nausea**						
Acute	98.5	93.4	0.050	87.3	87.9	0.747
Delayed	90.4	80.9	0.027	76.9	71.3	0.014
Overall	89.6	79.4	0.021	74.6	69.1	0.020
**Complete protection**						
Acute	97.0	87.5	0.006	82.3	81.1	0.528
Delayed	84.4	73.5	0.027	67.3	60.3	0.005
Overall	83.0	69.9	0.010	63.8	57.9	0.020

^1^ *P* value from logistic regression versus oral palonosetron; not adjusted for multiple comparisons.

^2^ *P* value from two-sided Cochran-Mantel-Haenszel test including treatment, age class, and region as strata.

HEC: highly emetogenic chemotherapy; AC: anthracycline cyclophosphamide; NEPA: netupitant/palonosetron; PALO: palonosetron; DEX: dexamethasone.

**Table 4:** Efficacy of NEPA in gender/age emetic risk groups.

Overall (0–120 h) CR % of patients	NEPA + DEX	Oral PALO + DEX	% Difference (95% CI)
**Females <55 years** (high risk) (*N* = 100/100)	80.0	69.0	11.0 (−1.0; 23.0)
**Females ≥55 years** (moderate risk) (*N* = 108/103)	84.5	69.4	15.0 (3.9; 26.2)
**Males <55 years** (low risk) (*N* = 91/126)	89.7	71.4	18.3 (7.6; 29.0)
**Males ≥55 years** (lowest risk) (*N* = 206/153)	92.8	81.1	11.7 (5.0; 18.5)

NEPA: netupitant/palonosetron; PALO: palonosetron; CR: complete response; CI: confidence interval.

**Table 5:** Efficacy of NEPA in older patients.

% of patients	Study 1 (HEC)		Study 2 (AC)		Study 3 (non-AC MEC/HEC)	
Time period	≥65 yrs (*N* = 20)	Overall population (*N* = 135)	≥65 yrs (*N* = 116)	Overall population (*N* = 724)	≥65 yrs (*N* = 78)	Overall population (*N* = 309)
Acute (0–24 h)	100	98.5	94.0	88.4	97.4	92.9
Delayed (25–120 h)	100	90.4	81.0	76.9	80.8	83.2
Overall (0–120 h)	100	89.6	79.3	74.3	78.2	80.6

HEC: highly emetogenic chemotherapy; AC: anthracycline cyclophosphamide; MEC: moderately emetogenic chemotherapy.

**Table 6:** Overview of adverse events.

Number (%) of patients with the following	Cycle 1			All cycles^*^		
	NEPA + DEX (*N* = 1442)	IV or oral PALO + DEX (*N* = 1600)	APR + OND/PALO + DEX (*N* = 238)	NEPA + DEX (*N* = 1033)	Oral PALO + DEX (*N* = 725)	APR + oral PALO + DEX (*N* = 104)
Any treatment-emergent AE (TEAE)	944 (65.5%)	945 (59.1%)	135 (56.7%)	1127 (78.2%)	1080 (67.5%)	166 (69.7%)

Treatment-related AE (TRAE)	138 (9.6%)	105 (6.6%)	29 (12.2%)	194 (13.5%)	134 (8.4%)	32 (13.4%)

Serious TEAE	33 (2.3%)	87 (5.4%)	4 (1.7%)	87 (6.0%)	99 (6.2%)	19 (8.0%)

Serious TRAE	2 (0.1%)	2 (0.1%)	—	3 (0.2%)	2 (0.1%)	—

TEAE leading to death	8 (0.6%)	20 (1.3%)	—	17 (1.2%)	21 (1.3%)	1 (0.4%)

TEAE leading to discontinuation	14 (1.0%)	6 (0.4%)	4 (1.7%)	44 (3.1%)	20 (1.3%)	13 (5.5%)

TRAE leading to discontinuation	2 (0.1%)	2 (0.1%)	—	1 (0.1%)	4 (0.3%)	—

^*^All cycles: Cycle 1 from all Phase 2/3 studies + Cycles 2 and beyond from the Phase 3 multiple cycle trials.

Treatment-emergent adverse event (TEAE): any AE reported after first study drug intake.

TRAE: AEs deemed possibly, probably, or definitely related to study drug.

DEX: dexamethasone, PALO: palonosetron, and APR: aprepitant.

## References

[1] Frame D. G. (2010). Best practice management of CINV in oncology patients: I. Physiology and treatment of CINV. Multiple neurotransmitters and receptors and the need for combination therapeutic approaches. *The Journal of Supportive Oncology*.

[2] Feyer P., Jordan K. (2011). Update and new trends in antiemetic therapy: the continuing need for novel therapies. *Annals of Oncology*.

[3] Roila F., Herrstedt J., Aapro M. (2010). Guideline update for MASCC and ESMO in the prevention of chemotherapy-and radiotherapy-induced nausea and vomiting: results of the Perugia consensus conference. *Annals of Oncology*.

[4] Basch E., Prestrud A. A., Hesketh P. J. (2011). Antiemetics: American Society of Clinical Oncology clinical practice guideline update. *Journal of Clinical Oncology*.

[5] (2014). *NCCN Clinical Practice Guidelines in Oncology (NCCN Guidelines)*.

[6] Aapro M., Molassiotis A., Dicato M. (2012). The effect of guideline-consistent antiemetic therapy on chemotherapy-induced nausea and vomiting (CINV): the Pan European Emesis Registry (PEER). *Annals of Oncology*.

[7] Gilmore J. W., Peacock N. W., Gu A. (2014). Antiemetic guideline consistency and incidence of chemotherapy-induced nausea and vomiting in US community oncology practice: INSPIRE study. *Journal of Oncology Practice*.

[8] Burmeister H., Aebi S., Studer C., Fey M. F., Gautschi O. (2012). Adherence to ESMO clinical recommendations for prophylaxis of chemotherapy-induced nausea and vomiting. *Supportive Care in Cancer*.

[9] Tendas A., Sollazzo F., Niscola P. (2013). Adherence to recommendation for chemotherapy-induced nausea and vomiting prophylaxis: the proposal of a score. *Supportive Care in Cancer*.

[10] Rojas C., Raje M., Tsukamoto T., Slusher B. S. (2014). Molecular mechanisms of 5-HT_3_ and NK_1_ receptor antagonists in prevention of emesis. *European Journal of Pharmacology*.

[11] Stathis M., Pietra C., Rojas C., Slusher B. S. (2012). Inhibition of substance P-mediated responses in NG108-15 cells by netupitant and palonosetron exhibit synergistic effects. *European Journal of Pharmacology*.

[12] Gralla R., Lichinitser M., van der Vegt S. (2003). Palonosetron improves prevention of chemotherapy-induced nausea and vomiting following moderately emetogenic chemotherapy: results of a double-blind randomized phase III trial comparing single doses of palonosetron with ondansetron. *Annals of Oncology*.

[13] Saito M., Aogi K., Sekine I. (2009). Palonosetron plus dexamethasone versus granisetron plus dexamethasone for prevention of nausea and vomiting during chemotherapy: a double-blind, double-dummy, randomised, comparative phase III trial. *The Lancet Oncology*.

[14] Aapro M. S., Grunberg S. M., Manikhas G. M. (2006). A phase III, double-blind, randomized trial of palonosetron compared with ondansetron in preventing chemotherapy-induced nausea and vomiting following highly emetogenic chemotherapy. *Annals of Oncology*.

[15] Jordan K., Sippel C., Schmoll H.-J. (2007). Guidelines for antiemetic treatment of chemotherapy-induced nausea and vomiting: past, present, and future recommendations. *The Oncologist*.

[16] Jordan K., Gralla R., Jahn F., Molassiotis A. (2014). International antiemetic guidelines on chemotherapy induced nausea and vomiting (CINV): content and implementation in daily routine practice. *European Journal of Pharmacology*.

[17] Jordan K., Kinitz I., Voigt W., Behlendorf T., Wolf H.-H., Schmoll H.-J. (2009). Safety and efficacy of a triple antiemetic combination with the NK-1 antagonist aprepitant in highly and moderately emetogenic multiple-day chemotherapy. *European Journal of Cancer*.

[18] dos Santos L. V., Lima J. P. (2014). Important clinical findings for chemotherapy-induced nausea and vomiting: commentary on molassiotis et al. *Journal of Pain and Symptom Management*.

[19] Schmidt N., Ricarte C., Haas G. (2014). Evaluation of treatment patterns in acute nausea and vomiting in EU5 countries. *Annals of Oncology*.

[20] Grunberg S. M. (2009). Obstacles to the implementation of antiemetic guidelines. *Journal of the National Comprehensive Cancer Network*.

[21] Kaiser R. (2005). Antiemetic guidelines: are they being used?. *The Lancet Oncology*.

[22] Cabana M. D., Rand C. S., Powe N. R. (1999). Why don’t physicians follow clinical practice guidelines? A framework for improvement. *The Journal of the American Medical Association*.

[23] Mertens W. C., Higby D. J., Brown D. (2003). Improving the care of patients with regard to chemotherapy-induced nausea and emesis: the effect of feedback to clinicians on adherence to antiemetic prescribing guidelines. *Journal of Clinical Oncology*.

[24] Dranitsaris G., Leung P., Warr D. (2001). Implementing evidence based antiemetic guidelines in the oncology setting: results of a 4-month prospective intervention study. *Supportive Care in Cancer*.

[25] Nolte M. J., Berkery R., Pizzo B. (1998). Assuring the optimal use of serotonin antagonist antiemetics: the process for development and implementation of institutional antiemetic guidelines at Memorial Sloan-Kettering Cancer Center. *Journal of Clinical Oncology*.

[26] Roila F. (2004). Transferring scientific evidence to oncological practice: a trial on the impact of three different implementation strategies on antiemetic prescriptions. *Supportive Care in Cancer*.

[27] Spinelli T., Calcagnile S., Giuliano C. (2014). Netupitant PET imaging and ADME studies in humans. *Journal of Clinical Pharmacology*.

[28] Thomas A. G., Stathis M., Rojas C., Slusher B. S. (2014). Netupitant and palonosetron trigger NK_1_ receptor internalization in NG108-15 cells. *Experimental Brain Research*.

[29] Calcagnile S., Lanzarotti C., Rossi G., Henriksson A., Kammerer K. P., Timmer W. (2013). Effect of netupitant, a highly selective NK_1_ receptor antagonist, on the pharmacokinetics of palonosetron and impact of the fixed dose combination of netupitant and palonosetron when coadministered with ketoconazole, rifampicin, and oral contraceptives. *Supportive Care in Cancer*.

[30] Lanzarotti C., Rossi G. (2013). Effect of netupitant, a highly selective NK1 receptor antagonist, on the pharmacokinetics of midazolam, erythromycin, and dexamethasone. *Supportive Care in Cancer*.

[31] Giuliano C., Lovati E., Funk C. (2012). In vitro drug-drug interaction studies with the antiemetic drug netupitant and its major metabolites (M1 and M2), involving main human cytochrome P450 isoenzymes. *Annals of Oncology*.

[32] Aapro M. S., Walko C. M. (2010). Aprepitant: drug-drug interactions in perspective. *Annals of Oncology*.

[33] Hesketh P. J., Rossi G., Rizzi G. (2014). Efficacy and safety of NEPA, an oral combination of netupitant and palonosetron, for prevention of chemotherapy-induced nausea and vomiting following highly emetogenic chemotherapy: a randomized dose-ranging pivotal study. *Annals of Oncology*.

[34] Aapro M., Rugo H., Rossi G. (2014). A randomized phase III study evaluating the efficacy and safety of NEPA, a fixed-dose combination of netupitant and palonosetron, for prevention of chemotherapy-induced nausea and vomiting following moderately emetogenic chemotherapy. *Annals of Oncology*.

[35] Gralla R. J., Bosnjak S. M., Hontsa A. (2014). A phase III study evaluating the safety and efficacy of NEPA, a fixed-dose combination of netupitant and palonosetron, for prevention of chemotherapy-induced nausea and vomiting over repeated cycles of chemotherapy. *Annals of Oncology*.

[37] Aapro M., Gralla R., Karthaus M. (2014). Multicycle efficacy and safety of NEPA, a fixed-dose antiemetic combination of netupitant and palonosetron, in patients receiving chemotherapy of varying emetogenicity. *Annals of Oncology*.

[38] National Institutes of Health https://clinicaltrials.gov/ct2/show/NCT01363479.

[39] Hesketh P. J., Jordan K., Gralla R. (2014). Prevention of chemotherapy-induced nausea and vomiting with a fixed-dose combination of netupitant and palonosetron (NEPA) following highly emetogenic chemotherapy: evaluation of response based on gender and age. *Annals of Oncology*.

[40] Sheehy C. M., Perry P. A., Cromwell S. L. (1999). Dehydration: biological considerations, age-related changes, and risk factors in older adults. *Biological Research for Nursing*.

[41] Manson A., Shea S. (1991). Malnutrition in elderly ambulatory medical patients. *American Journal of Public Health*.

[42] Aapro M., Hesketh P. J., Gralla R. J., Jordan K., Rizzi G. (2014). Safety and efficacy of NEPA, a fixed oral dose combination of netupitant and palonosetron, in older patients. *Journal of Geriatric Oncology*.

[43] Gralla R. J., de Wit R., Herrstedt J. (2005). Antiemetic efficacy of the neurokinin-1 antagonist, aprepitant, plus a 5HT_3_ antagonist and a corticosteroid in patients receiving anthracyclines or cyclophosphamide in addition to high-dose cisplatin: analysis of combined data from two phase III randomized clinical trials. *Cancer*.

[44] Hesketh P. J., Gralla R., Rossi G. (2014). NEPA, a fixed-dose antiemetic combination of netupitant and palonosetron: results of effectiveness in 407 patients receiving cisplatin plus chemotherapy of various emetic risk. *Support Care Cancer*.

[45] Jordan K., Gralla R., Rossi G. (2014). Is the addition of an NK_1_ receptor antagonist beneficial in patients receiving carboplatin? Supplementary data with NEPA, a fixed-dose combination of netupitant and palonosetron. *Supportive Care in Cancer*.

[46] Aapro M., Karthaus M., Schwartzberg L. (2014). Phase 3 study of NEPA, a fixed-dose combination of netupitant and palonosetron, for prevention of chemotherapy-induced nausea and vomiting during repeated moderately emetogenic chemotherapy (MEC) cycles. *Journal of Clinical Oncology*.

[47] Aapro M., Hesketh P. J., Jordan K. (2014). Safety of NEPA, an oral fixed-dose combination of netupitant and palonosetron: pooled data from the phase 2/3 clinical program. *Support Care Cancer*.

[48] Morganroth J., Parisi S., Moresino C., Thorn M., Cullen M. T. (2007). High dose palonosetron does not alter ECG parameters, including QTc interval in healthy subjects: results of a dose-response, double blind, randomized, parallel El4 study of palonosetron vs. moxifloxacin or placebo. *European Journal of Cancer Supplements*.

[49] Spinelli T., Moresino C., Baumann S., Timmer W., Schultz A. (2014). Effects of combined netupitant and palonosetron (NEPA), a cancer supportive care antiemetic, on the ECG of healthy subjects: an ICH E14 thorough QT trial. *SpringerPlus*.

[50] Gonullu G., Demircan S., Demirag M. K., Erdem D., Yucel I. (2012). Electrocardiographic findings of palonosetron in cancer patients. *Supportive Care in Cancer*.

[51] Yavas C., Dogan U., Yavas G., Araz M., Yavas Ata O. (2012). Acute effect of palonosetron on electrocardiographic parameters in cancer patients: a prospective study. *Supportive Care in Cancer*.

[52] Dogan U., Yavas G., Tekinalp M., Yavas C., Ata O. Y., Ozdemir K. (2012). Evaluation of the acute effect of palonosetron on transmural dispersion of myocardial repolarization. *European Review for Medical and Pharmacological Sciences*.

[53] Rapoport B. L., Jordan K., Boice J. A. (2010). Aprepitant for the prevention of chemotherapy-induced nausea and vomiting associated with a broad range of moderately emetogenic chemotherapies and tumor types: a randomized, double-blind study. *Supportive Care in Cancer*.

[54] Warr D. G., Hesketh P. J., Gralla R. J. (2005). Efficacy and tolerability of aprepitant for the prevention of chemotherapy-induced nausea and vomiting in patients with breast cancer after moderately emetogenic chemotherapy. *Journal of Clinical Oncology*.

[55] Poli-Bigelli S., Rodrigues-Pereira J., Carides A. D. (2003). Addition of the neurokinin 1 receptor antagonist aprepitant to standard antiemetic therapy improves control of chemotherapy-induced nausea and vomiting: results from a randomized, double-blind, placebo-controlled trial in Latin America. *Cancer*.

[56] Hesketh P. J., Grunberg S. M., Gralla R. J. (2003). The oral neurokinin-1 antagonist aprepitant for the prevention of chemotherapy-induced nausea and vomiting: a multinational, randomized, double-blind, placebo-controlled trial in patients receiving high-dose cisplatin—the Aprepitant Protocol 052 Study Group. *Journal of Clinical Oncology*.

[57] Schmoll H. J., Aapro M. S., Poli-Bigelli S. (2006). Comparison of an aprepitant regimen with a multiple-day ondansetron regimen, both with dexamethasone, for antiemetic efficacy in high-dose cisplatin treatment. *Annals of Oncology*.

[58] Rapoport B. L. (2014). Efficacy of a triple antiemetic regimen with aprepitant for the prevention of chemotherapy-induced nausea and vomiting: effects of gender, age, and region. *Current Medical Research and Opinion*.

[59] Gralla R., Rapoport B., Brown C., Street J. C., Hardwick J. S., Carides A. D. (2009). 57LBA Aprepitant (APR) for the prevention of chemotherapy-induced nausea and vomiting (CINV) associated with moderately emetogenic chemotherapy (MEC) in breast and non-breast cancers. *European Journal of Cancer Supplements*.

[60] Yahata H., Sonoda K., Kobayashi H. (2014). Aprepitant for the prevention of chemotherapy-induced nausea and vomiting with a moderately emetogenic chemotherapy: a multicenter, placebo-controlled, double-blind, randomized study in Japanese gynecologic patients receiving paclitaxel and carboplatin. *Annals of Oncology*.

[61] Schnadig I., Modiano M. R., Poma A. (2014). Phase 3 trial results for rolapitant, a novel, NK-1 receptor antagonist, in the prevention of chemotherapy-induced nausea and vomiting (CINV) in subjects receiving moderately emetogenic chemotherapy (MEC). *Journal of Clinical Oncology*.

[62] US Food and Drug Administration (FDA) (2011). *FDA Drug Safety Communication: Abnormal Heart Rhythms May Be Associated with Use of Zofran (Ondansetron)*.

[63] Food and Drug Administration (2010). *FDA Drug Safety Communication: Abnormal Heart Rhythms Associated with Use of Anzemet (Dolasetron Mesylate)*.

[64] FDA Safety (2009). *Kytril (Granisetron Hydrochloride) Injection, Tablets and Oral Solution*.

[65] Schwartzberg L., Oprean C., Cardona-Huerta S. (2013). No evidence of increased cyclophosphamide toxicity associated with the antiemetic agent NEPA, a fixed-dose combination of netupitant and palonosetron. *Blood*.

[66] Karthaus M., Aapro M., Rizzi G. (2014). Cardiac safety of NEPA, a fixed-dose antiemetic combination, administered prior to anthracycline-based chemotherapy. *Blood*.

